# RNA-editing-mediated exon evolution

**DOI:** 10.1186/gb-2007-8-2-r29

**Published:** 2007-02-27

**Authors:** Galit Lev-Maor, Rotem Sorek, Erez Y Levanon, Nurit Paz, Eli Eisenberg, Gil Ast

**Affiliations:** 1 Department of Human Molecular Genetics and Biochemistry, Sackler Faculty of Medicine, Tel Aviv University, Ramat Aviv 69978, Israel; 2 Genomics Division, Lawrence Berkeley National Laboratory, Berkeley, CA 94720, USA; 3 Compugen Ltd, Pinchas Rosen St, Tel-Aviv 69512, Israel; 4 Department of Genetics, Harvard Medical School, Avenue Louis Pasteur, Boston, Massachusetts 02115, USA; 5 Department of Pediatric Hemato-Oncology, Chaim Sheba Medical Center, Sackler Faculty of Medicine, Tel Aviv University, Ramat Aviv 69978, Israel; 6 School of Physics and Astronomy, Raymond and Beverly Sackler Faculty of Exact Sciences, Tel-Aviv University, Ramat Aviv 69978, Israel

## Abstract

**Background:**

*Alu *retroelements are specific to primates and abundant in the human genome. Through mutations that create functional splice sites within intronic *Alu*s, these elements can become new exons in a process denoted exonization. It was recently shown that *Alu *elements are also heavily changed by RNA editing in the human genome.

**Results:**

Here we show that the human nuclear prelamin A recognition factor contains a primate-specific *Alu*-exon that exclusively depends on RNA editing for its exonization. We demonstrate that RNA editing regulates the exonization in a tissue-dependent manner, through both the creation of a functional AG 3' splice site, and alteration of functional exonic splicing enhancers within the exon. Furthermore, a premature stop codon within the *Alu*-exon is eliminated by an exceptionally efficient RNA editing event. The sequence surrounding this editing site is important not only for editing of that site but also for editing in other neighboring sites as well.

**Conclusion:**

Our results show that the abundant RNA editing of *Alu *sequences can be recruited as a mechanism supporting the birth of new exons in the human genome.

## Abstract 2

A primate-specific exon is found to be dependent on RNA editing for its exonization.

## Background

Analysis of the sequenced human genome has revealed that it contains about 200,000 exons [1]. However, the exon content in mammalian genes is far from static. Rather, it is constantly changing through a dynamic evolutionary process in which exons are newly created and deleted. New exons can arise from gene duplication [2] and exon duplication [3], but perhaps the most intriguing process by which exons can be born is exonization by exaptation, where genomic sequences that did not originally function as exons are adopted into exonic sequences [4].

We have recently shown that *Alu *elements use this exonization mechanism to give rise to hundreds of novel internal exons in the human genome [5]. *Alu *elements are unique primate-specific retrotransposons that occur in over one million copies in the human genome [2,6,7]. Their 300 bases-long consensus sequence contains motifs that resemble 5' and 3' potential splice sites (5' ss and 3' ss, respectively). Random mutations can turn these motifs into functional splice sites that can be recognized by the splicing machinery [5,8,9]. *Alu*-derived internal exons are almost always alternatively spliced, allowing the original isoform to coexist with the new one and preventing the deleterious effects of introducing a new protein at the expense of the original one [5,8]. Thus, *Alu *elements can increase the coding capacity of human genes while maintaining the original protein repertoire.

Recently, *Alu*s were reported to contribute to human transcriptome diversity by an additional mechanism, involving adenosine-to-inosine (A-to-I) RNA editing. A-to-I RNA editing refers to the deamination of selected adenosine residues, altering the nucleotide sequence of RNA transcripts from that encoded by genomic DNA. It is catalyzed by enzymes from the ADAR (adenosine deaminase acting on RNA) family. Editing targets are typically located within double stranded RNA (dsRNA), which is recognized by the ADAR enzymes [10]. RNA editing can cause non-synonymous codon changes when occurring inside the coding sequence or occur in the non-coding parts of the pre-RNA molecule. It was lately reported that human transcripts contain excess editing over mouse, rat, chicken and fly transcripts [5,11,12]. The majority of editing sites in human (approximately 96%) were found to occur within *Alu *sequences. Due to the abundance of *Alu *elements in the human genome, two *Alu*s in opposite orientation are frequently found near each other. When transcribed in the pre-mRNA, these two *Alu*s can presumably fold to create a dsRNA; this substrate is recognized and edited by the ADAR enzymes. Edited *Alu*s typically do not contribute to the protein repertoire, but rather reside in non-coding parts of the pre-RNA molecule - untranslated regions (UTRs) and introns [11,13-18].

The observation that *Alu *elements are both extensively edited and can give rise to novel alternatively spliced exons in primate genomes raises the question of whether RNA editing can be involved in the birth of new *Alu*-exons. RNA editing was previously shown to regulate alternative splicing by creating a splice site [19]. The most studied event is the auto-editing of the *ADAR2 *gene, in which intronic AA dinucleotide turns into a functional AG 3' ss following RNA editing [19]. Indeed, it was recently suggested that such editing events may create functional splice sites in silent intronic *Alu *elements, thus promoting their exonization [13]. In this study we detected a novel primate-specific *Alu*-exon that exclusively depends on RNA editing for its exonization. We show that RNA editing regulates the exonization in a tissue-dependent manner, both through creation of a functional AG 3' ss, elimination of a premature stop codon, and regulation of the inclusion/skipping level through alteration of exonic splicing enhancers and silencers within the exon. We also demonstrated that the sequence around an editing site is important not only for the editing in that site but also editing in neighboring sites located along this *Alu*-exon. Our results show that RNA editing can be recruited as a mechanism supporting the birth of new exons in the human genome.

## Results

### RNA editing enables exonization of a nuclear prelamin A recognition factor *Alu*-exon

To check the possibility that *Alu*-derived exons were fixed into protein-coding genes through an RNA-editing-mediated process, we first used expressed sequence tags (ESTs) and cDNAs from GenBank version 136 aligned to the human genome (version hg16) to identify internal human exons that contain *Alu *elements, as described in [5]. We looked for *Alu*-containing exons flanked by either AA at the 3' ss or AT at the 5'ss. These non-canonical splice sites will normally not be selected by the splicing machinery; however, each of these splice sites is theoretically capable of becoming a *bona fide *splice site through A-to-I RNA editing, because inosine is recognized by the splicing apparatus as a guanosine [19]. We demanded that the *Alu*-exon will be supported by more than one EST/cDNA, and that it neither induced frame-shift nor contained a stop codon, as these parameters were shown to be indicative of functional alternatively spliced exons [5].

**Figure 1:**
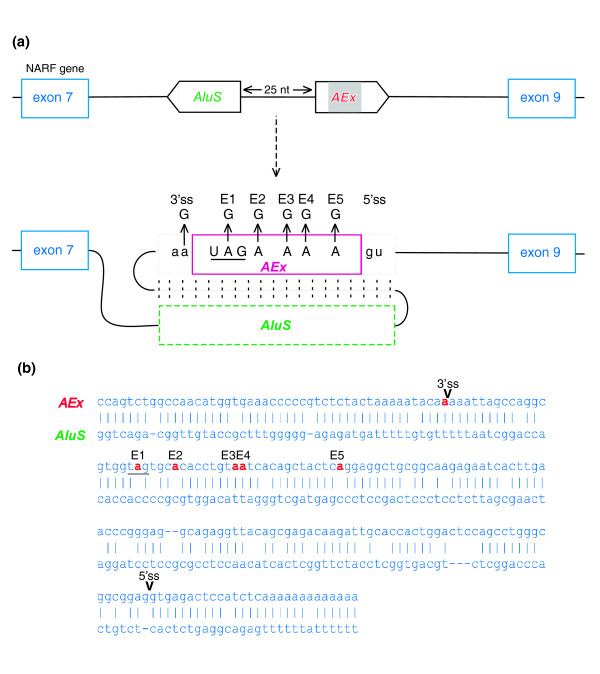
The birth of an *Alu*-exon through RNA editing. Editing prediction was inferred from alignment of cDNAs to human genomic DNA. **(a) **Schematic illustration of exons 7 to 9 of the *NARF *gene. Exons are depicted as blue boxes; the *Alu*-exon, derived from *Alu*Sx (AEx; purple box), is in a sense orientation and is shown in the middle. The intronic, antisense-orientation *Alu *sequence (*Alu*S) is 25 base-pairs upstream of the exonized *Alu*. Sense and antisense *Alu*s are expected to create a dsRNA secondary structure, thus allowing RNA editing. RNA editing changes an AA dinucleotide into a functional AG 3' splice site (lower panel). RNA editing also occurs in five positions in the *Alu*-exon itself (E1, E2, E3, E4 and E5). In the first position (E1), editing changes a UAG stop codon into a UGG Trp codon. **(b) **Predicted folding between the sense and antisense *Alu *sequences (upper and lower lines, respectively). Adenosines that undergo editing are marked by red. Splice sites utilized for *Alu *exonization are marked as 5' ss and 3' ss on the alignment.

We were able to detect one such *Alu*-derived exon, having an AA 3' ss, that conformed to the above conditions (Figure 1). This exon is the eighth exon in the nuclear prelamin A recognition factor (NARF), a protein that interacts with the carboxyl-terminal tail of prelamin A and localizes to the nuclear lamina [20]. As with all internal *Alu*-containing exons described to date, it is alternatively spliced, and exon-inclusion is supported by seven cDNAs, including the full-length cDNA (GenBank:BC000438; UCSC March 2006 version). The exon inserts 46 in-frame additional amino acids into the coding sequence of NARF.

A-to-I RNA editing takes place in the context of dsRNA, to which the ADAR proteins bind through a dsRNA-binding domain (reviewed in [21,22]). It was therefore shown that, for the vast majority of edited *Alu*s in human exons, there is a nearby (up to 2,000 base-pairs apart) intronic *Alu *counterpart in the opposite orientation, which presumably serves as the template for dsRNA formation [13,14]. Indeed, we were able to find an oppositely oriented *Alu *sequence 25 bases upstream of the exonized *Alu*. The antisense *Alu *has 81% identity when aligned to the exon-producing *Alu*, suggesting that these two *Alu*s might form a stable intramolecular dsRNA formation following transcription (Figure 1b). This, in addition to the non-canonical AA splice site, implies that RNA editing participates in the exonization of that *Alu*.

Editing within *Alu *elements frequently occurs in more than one position, due to the long RNA duplex usually formed by two oppositely oriented nearby *Alu*s [11,13-15,18,23]. Indeed, by searching for A→G discrepancies in the alignments of cDNAs to the human genome we detected five additional potential editing sites in the *Alu*-exon (Figure 1b, E1-E5). The first of these, found in position 19 of the exon, is of particular interest, because it has the potential to change a TAG codon (termination of translation) to a TGG codon (coding for tryptophane). In the absence of RNA editing in the E1 position, the insertion of this *Alu*-exon would have caused a premature termination. It is important to note that the editing in the exonic E1-E5 sites is directly recognizable from the ESTs or cDNAs in comparison to human genomic DNA, whereas editing in the potential 3'ss is postulated based only on the genomic sequence.

### Different levels of exonization among human tissues

**Figure 2:**
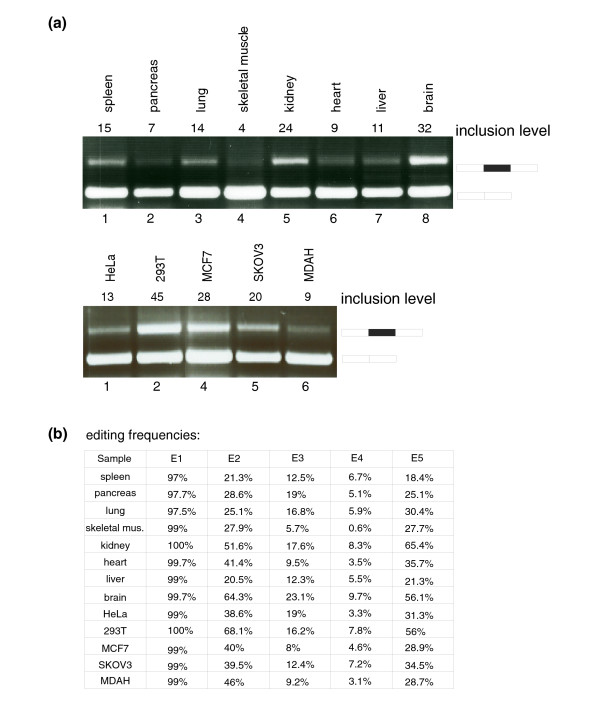
Levels of *Alu*-exon inclusion and RNA editing in the endogenous human *NARF *gene. **(a) **cDNAs from various normal human tissues or cDNAs from various cell-lines were PCR amplified using primers specific for the two exons flanking the exonized *Alu *(upper and lower panels, respectively). The inclusion level of the *Alu*-exon is indicated at the top of the panel, and represents the total percentage of the *Alu*-containing mRNA isoform, where 100% corresponds to the total of both mRNA isoforms (inferred by the ImageJ program). Each PCR product was confirmed by sequencing. Schemata of the two mRNA products are shown on the right. **(b) **Editing efficiency in the five exonic sites (E1, E2, E3, E4 and E5; see Figure 1 for site positions) in different tissues and cell lines. The editing frequencies in each of the five edited sites, derived from sequence results obtained from an average of three independent amplifications, were quantified using the Discovery Studio Gene 1.5 program.

To check whether this putative *Alu*-exon is indeed spliced into the mature mRNA of NARF, we tested the existence of the exon-inclusion and exon-skipping forms in endogenous mRNA from various normal human tissues and cell-lines. As shown in Figure 2, the inclusion form was detected in all cDNAs generated from normal human tissues as well as from different human cell lines. This indicates that the exonization of the NARF *Alu *is evolutionarily fixed in the human transcriptome. Moreover, exon inclusion levels in different tissues followed expected levels of RNA editing in those tissues. For example, brain, kidney and spleen showed the highest levels of exon inclusion while skeletal muscle showed the lowest levels of exon inclusion (Figure 2a; Additional data file 1). These results are in line with genome wide analysis of edited RNA in different tissues [5,11,13,15] and to the amount of inosine detected in RNA in various tissues [24]. The above results further suggest that RNA editing is involved in the regulation of alternative splicing of the exonized *Alu *in the gene encoding NARF. Interestingly, we note high levels of exon inclusion in MCF7 and 293T cell-lines, but not in HeLa, SKOV3 and MDAH cell-lines (human cancer cell lines originated from breast, kidney, cervix and ovaries, respectively) (Figure 2a), although the global editing level in cell-lines is expected to be relatively low [11]. This demonstrates that the amplitude of editing level in various cell line types is of a variable nature.

We sequenced all of the exon inclusion PCR products and analyzed the editing frequencies at the five editing sites (named E1-E5, Figure 1b) using the Discovery Studio (DS) Gene 1.5 program (Accelrys Inc., San Diego, CA, USA). Importantly, the first exonic editing site, E1 (at position 19 of the exon), was edited at nearly 100% efficiency in all tested tissues and cell lines, whereas the editing levels of the other sites varied (E2 being edited in an average of 53.6% of RNAs, E3 in an average of 26.1%, E4 in an average of 7.9% and E5 in an average of 37.5%) (Figure 2b). Notably, editing sites E1, E3 and E5 are mistakenly annotated as single nucleotide polymorphisms (SNPs) in dbSNP [25] (rs17855348, rs17849311 and rs17855349, respectively) based on the variance in cDNA data. Many similar examples of RNA editing sites erroneously deposited in dbSNP have been recently reported [26].

Usually, editing efficiency is much lower than 100% per site, depending on the expression levels of the ADAR enzymes in the given tissue, the secondary structure of the substrate, or the surrounding sequence. As shown above, position E1 in the NARF *Alu*-exon is edited in nearly all RNA molecules containing this exon. Inactivation of the nonsense-mediated mRNA decay (NMD) by adding puromycine (see Materials and methods) to 293T cell line did not affect the >97% editing efficiency in site E1 (data not shown). This indicates that the high level of editing in site E1 is not due to elimination of unedited, stop-codon containing mRNAs, but rather is indicative of a high efficiency of editing in that site. Apart from the Q/R site of gluR-B [27], which is restricted to brain, this is the highest editing efficiency documented in human, though it has a much broader tissue expression spectrum. This result suggests that additional regulatory mechanisms have evolved to ensure that the stop codon is edited to a Trp codon in all mRNAs containing the *Alu*-exon. It further implies that the exonization of the NARF *Alu*-exon is functional in the human transcriptome.

### *Alu*-*Alu *dsRNA directs exonization

**Figure 3:**
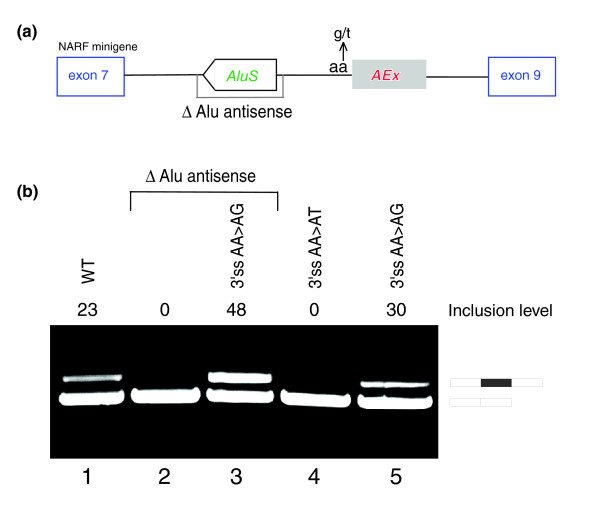
The antisense *Alu *is essential for exonization. **(a) **An illustration of the *NARF *minigene that was constructed, containing the genomic sequence of the human *NARF *gene from exon 7 to 9. The sites that were mutated in (b) are shown. **(b) **The minigene was transfected to human 293T cells, and total RNA was collected and examined by RT-PCR analysis using specific primers to mRNA products of the plasmid minigene. The first lane is the wild-type (WT) pattern. Lanes 2 and 3 represent a deletion of the antisense intronic *Alu*. Lane 3 also represents an AA→AG mutation at the 3'ss. Lanes 4 and 5 represent an AA→AT and AA→AG mutation at the 3'ss (without deletion of the antisense *Alu*), respectively. The inclusion level of the *Alu*-exon is indicated at the top of the gel, and represents the total percentage of the edited-*Alu*-containing mRNA isoform, where 100% corresponds to the total of both mRNA isoforms (inferred using the ImageJ program). Schemata of the two mRNA products are shown on the right.

To substantiate the possibility that exon 8 in NARF was exonized through an RNA-editing-mediated process, we constructed a minigene containing the human genomic sequence of the gene encoding NARF from exon 7 to 9, including the two introns in between and the alternative *Alu*-exon. Following transfection of this minigene into 293T cells, total RNA was collected, and the splicing pattern of the NARF minigene was examined by RT-PCR analysis using primers specific to the plasmid cDNA and not the endogenous one (see Materials and methods). We then tested the effect of serial intronic and exonic mutations on the splicing of the *Alu*-exon (Figure 3a).

When the wild-type minigene was transfected into 293T cells, 23% of the mature mRNAs derived from this minigene represented the exon-inclusion form (Figure 3b, lane 1). However, deletion of the antisense *Alu *element upstream of the exonized *Alu *resulted in total abrogation of exon inclusion (Figure 3b, lane 2), indicating that these two adjacent *Alu*s probably pair to create the dsRNA that is required for RNA editing. Without this dsRNA, editing does not occur, and functional AG in the 3'ss cannot be created. The effect of the antisense *Alu *deletion was reversed when the AA splice site near the *Alu*-exon was mutated to AG, indicating that a single AA→AG change is sufficient for exonization of this *Alu *(Figure 3b, lane 3). Interestingly, the AA→AG mutation increased exon-inclusion two-fold over the wild type, suggesting that, in 293T cells, about one-third of the AA pairs in the 3'ss are edited into a functional AG 3' ss. Also, a single AA→AT mutation at the 3'ss, created on the wild-type plasmid, resulted in full exon skipping, indicating the importance of editing at that site for exonization. Whereas, a single AA→AG mutation resulted in approximately 30% exonization (Figure 3b, lanes 4 and 5). The higher level of exonization after a single AA→AG mutation at the 3'ss without and with the antisense *Alu *presumably suggests that although the antisense *Alu *is essential for exonization, it also reduces the level of maximum exonization by interfering with spliceosome accessibility to the *Alu*-exon due to dsRNA formation (compare lanes 3 and 5). Combined together, these results demonstrate that the exonization of the *Alu*-exon 8 in NARF is mediated by RNA editing, and that this mechanism also controls the level of inclusion of this exon in different tissues.

### Editing in one site affects the level of editing in other sites and the surrounding sequence and the opposite nucleotide are important for editing

**Figure 4:**
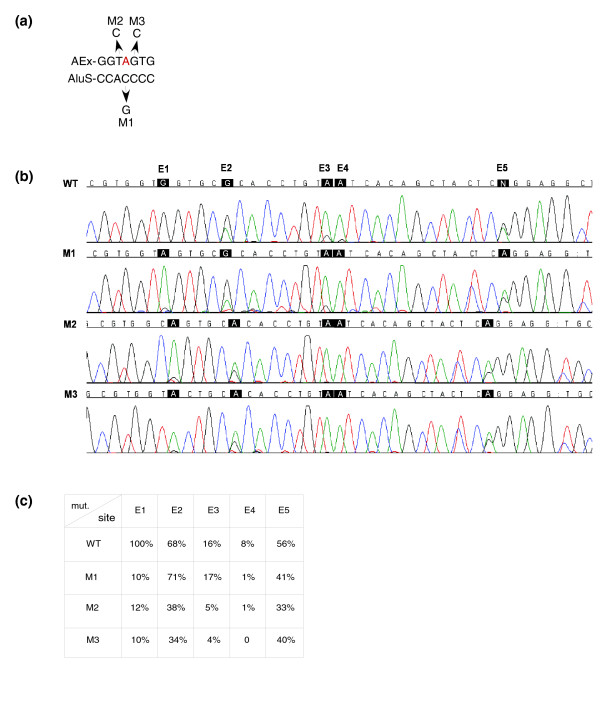
Editing is directed by a specific sequence surrounding the editing nucleotide. **(a) **An illustration showing the positions that were mutated in the *Alu*-exon (*AEx*) and the antisense intronic *Alu*S (*Alu*S): the flanking nucleotides of the edited E1 site, and the position in the antisense *Alu *that is predicted to be opposite to the E1 in the dsRNA formation. **(b) **Chromas sequences of the *Alu*-exon editing of the wild-type (WT) and three mutants from (a). WT and mutant plasmids were introduced into 293T cells by transfection, total RNA was extracted, and splicing products were separated on 1.5% agarose gel following RT-PCR analysis. The *Alu*-exon inclusion of the WT and mutants is highly similar (not shown). The edited positions are highlighted in black. **(c) **Rounded editing frequencies of each of the five edited sites, from three separate experiments, were quantified using the Discovery Studio Gene 1.5 program.

To test the possibility that specific sequences within the *Alu*-*Alu *duplex are involved in the regulation of high efficiency editing at the E1 site, we mutated the two nucleotides surrounding the edited site, as well as the nucleotide in the antisense *Alu *that is postulated to be opposite to the edited nucleotide within the dsRNA (see Figure 4a for the mutated nucleotides). All these mutations substantially reduced the editing in the E1 site (Figure 4b,c), indicating the importance of the surrounding sequence and the postulated opposite nucleotide in the antisense *Alu *for editing at that site. Moreover, mutations M2 and M3 also resulted in a significant reduction of RNA editing in the other exonic sites - the most significant effect was on site E2 (Figure 4b,c). This might suggest that the edited position is part of a sequence motif that directs high efficiency RNA editing at the other sites as well.

Our results indicate that RNA editing not only enables the exonization of the NARF *Alu*-exon, but also regulates its inclusion levels in different tissues (Figure 2). This regulation is probably attributed to the efficiency by which the AA splice site is edited to AG. However, another possible mechanism by which RNA editing can control exon inclusion levels is by altering exonic splicing enhancers and silencers (ESEs and ESSs, also denoted exonic splicing regulatory sequences (ESRs)) within the *Alu*-exon (Table 1). Indeed, editing of the first exonic site (E1) is predicted to eliminate a putative ESR ([28]; see also ESRsearch [29]. It also exchanges a putative ESS (GGTAGT) with another putative ESS (TGGTGG), as predicted by RescueESE [30]. In addition, the second exonic edited site (E2; position 30 in the exon) is part of four putative SR binding sites (Serine/Argenine-rich domain); editing reduces the score of the SF2/ASF binding site, eliminates a putative SRp40 ESR, creates a SRp55 ESR and also eliminates a putative ESR (as predicted by ESEfinder and ESRsearch [28,31]). In site E3, editing creates a putative high-scoring recognition site for the splicing factor SC35, as predicted by ESEfinder. Editing of E4 creates a putative recognition site for the splicing factor SC35, as predicted by ESEfinder. Editing of site E5 is predicted to have an effect on multiple ESRs (Table 1).

### RNA editing regulates the inclusion level of the NARF *Alu*-exon

**Figure 5:**
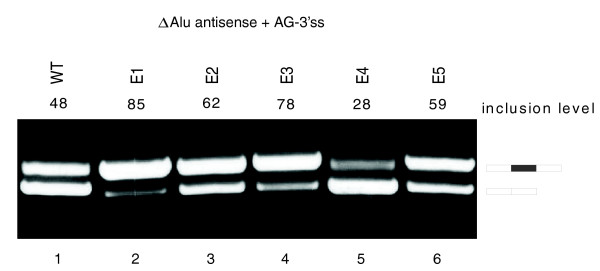
The effect of editing in exonic sites on exon inclusion levels. Lane 1 represents a deletion of the *Alu *antisense and also a mutation that creates an AG at the 3'ss. This plasmid was used to generate an A-to-G mutation in each of the exonic edited sites (lanes 2-6). This is a similar analysis to that shown in Figure 3.

To test the possibility that RNA editing regulates the inclusion levels of the NARF *Alu*-exon by altering ESRs within the exon, we serially mutated each of the exonic edited sites from A-to-G, simulating 100% editing efficiency. To examine the effect of the exonic sites only we used a minigene in which an A-to-G mutation mimics 100% editing in the 3'ss, and we also deleted the antisense *Alu *that affects editing of the exonic sites. As shown in Figure 5, an A-to-G mutation in E1 and E3, but not in E2 and E5, resulted in a significant increase in exon inclusion levels (Figure 5, compare lanes 2 and 4 with lanes 3 and 6). However, editing in position E4 significantly reduced the inclusion level, suggesting the creation of a putative SC35 site that functions as an ESS (Figure 5, lane 5). These results indicate that editing of three out of the five exonic edited sites affects alternative splicing levels. However, it is unlikely that alternative splicing is regulated through editing in the E1 site, because it is uniformly edited at high levels in all tissues tested (Figure 2b).

## Discussion

We have demonstrated that the NARF *Alu*-exon 8 is exonized via RNA editing and that RNA editing is also involved in its tissue-dependent regulation. Previously, RNA editing was implicated in both anti-viral protection and transcript diversity regulation; we now show that editing can also support evolutionary processes such as the birth of new exons. In a recent study, Athanasiadis *et al*. [13] presented computational predictions of several *Alu *exonization events (not including the NARF *Alu*-exon) that were hypothesized to be regulated by RNA editing; our results provide exemplary confirmation of the validity of these predictions.

It has been shown that a few hundred *Alu *elements become exonized through single base-pair mutations that create functional splice sites within their sequences. Yet in the case of the NARF *Alu*-exon, exonization strictly depends on RNA editing. This situation provides a simple, yet powerful, way to regulate the levels of exon inclusion in a tissue/developmental stage-specific manner. Since editing levels control the level of *Alu*-exon inclusion, exon inclusion rates would follow the varying editing levels in different tissues (Figure 2). Usually, many regulatory sequence elements are needed to regulate alternative splicing in a tissue-specific manner. These sequence elements presumably can extend up to 150 bases from each side of the regulated exon [32]. It is unlikely that a recently retroposed *Alu *element will carry all needed splicing regulatory elements; however, the RNA-editing-dependent exonization does not rely on such extensive sequence elements, and mainly depends on the expression level of the editing enzymes (ADARs) in the specific tissue. Moreover, we show that editing of two out of the five exonic edited sites affects alternative splicing levels (Figure 5). This provides an additional layer of regulation of alternative splicing through RNA editing.

Interestingly, the E1 as well as the E5 editing sites in the rhesus macaque (but not in chimpanzee) genome encode 'G', thus presenting only the edited version of the gene in those sites. However, there are differences between the genomic sequence of human and chimpanzee and that of rhesus. The *Alu*-exon (*Alu*Sx) and the sequence upstream and downstream of it are highly conserved between human and chimpanzee. But in the rhesus macaque there was an insertion of *Alu*Y (in the sense orientation) immediately upstream of *Alu*Sx (the one that exonized in human), leading the antisense *Alu*Sg (the one that forms the dsRNA) to be located 344 nucleotides upstream of the sense *Alu*Sx (and not 25 nucleotides upstream as it is in human). In addition, there was an insertion of 8 nucleotides in the sense *Alu*Sx in the rhesus macaque as well as a deletion of 44 nucleotides that includes the site used in human as 3'ss (Additional data file 2). These differences raise the question of whether *Alu*Sx in the rhesus macaque exonized at all.

The observed exonization of the NARF *Alu*-exon in all tested tissues and cell lines indicates that this exon is a *bona fide*, fixed functional exon in the human genome that originated from an exapted *Alu *(that is, an *Alu *that adopted a new function that was not its original function) [4]. An additional example for such exaptation is exon 8 of the *ADAR2 *gene, which is an *Alu*-exon of 120 nucleotides (inserts 40 amino acids). The *Alu*-exon inclusion isoform does not change the specificity of ADAR2 activity compared to the original isoform (exon skipping) but rather changes the rate of the enzymatic activity [33].

Few mammalian ADAR substrates in which editing causes amino acid substitutions have been found so far; the first (and most studied ones) encode receptors that are all expressed in the central nervous system, including subunits of the glutamate receptor superfamily [27], the serotonin 5-HT2C-receptor [34] and the potassium channel KCNA1 [35]. In all these examples, the amino acid substitutions due to editing have been shown to have a major impact on protein properties, and altered editing patterns in the genes encoding them have been found to be associated with several diseases, such as epilepsy [36], depression [37], ALS (Amyotrophic Lateral Sclerosis) [38], and malignant gliomas [39]. Lately, additional evolutionarily conserved RNA editing sites that lead to a codon exchange have been discovered in another four genes [15,40] - the functional importance of these sites was deduced by their extreme evolutionary conservation. The editing in the NARF *Alu*-exon is the only experimentally verified editing site in the coding region that is primate-specific. It would be interesting, therefore, to understand the function of the *Alu*-containing NARF isoform in the human transcriptome (as it might be responsible for a primate-specific trait); however, as the function of NARF itself is currently not clear, this must await future studies. A Pfam analysis indicates that the *Alu*-exon is inserted in NARF within a domain defined as 'Iron only hydrogenase large subunit, carboxy-terminal domain', and hence can presumably affect the substrate binding affinity/specificity, or the catalytic activity, of this domain.

It is worth noting that several other editing targets that cause predicted amino acid changes were detected in a genome-wide search for editing in *Alu *[13,15], but most of them were located in predicted genes or in aberrantly spliced RNAs. Thus, the actual expression of proteins from these transcripts and the possible functional implications of these sites remain to be verified.

Our study provides additional verification for the close relationship between splicing and editing, which was demonstrated when physical association between spliceosomal components and ADAR proteins was reported [41]. The actual mechanism that controls the interconnection of splicing and editing is still largely unknown, but it was shown that additional nuclear machineries are involved, such as the carboxy-terminal domain of RNA polymerase II in the auto-editing of ADAR2 [42,43]. This auto-editing is so far the most studied demonstration of the feedback loop between editing and splicing, where editing-mediated inclusion of an exon fragment in the rat *ADAR2 *gene changes, in turn, the editing capacitates of the ADAR protein itself [19]. Editing-mediated selection of splice sites has also been observed in other genes [39,44]. ADAR2 knockout mice provide another example of the tight connection between editing and splicing, since the absence of editing in the Q/R site prevents proper splicing of the nearby intron [45]. Our results show that this splicing-editing interconnection can also have evolutionary significance.

Although several thousand *Alu *sequences have the potential to undergo exonization [5], we were able to detect only one reliable event of a coding *Alu*-exon that seemed to be exonized through RNA editing, indicating that such a combination of evolutionary events is relatively rare in the human genome. However, this evolutionary mechanism for the birth of new exons might recur in other genomes. Moreover, this mechanism might allow additional *Alu *exonizations in the evolutionary future of *Homo sapiens *and other primate species. As some *Alu*s are still active in the human genome (at a rate of 1 transposition every 200 births [46]), a novel *Alu *retroposition in the opposite orientation from a nearby preexisting *Alu *might lead to dsRNA formation and *Alu*-exonization even if this *Alu *does not contain a canonical splice site.

## Conclusion

We have shown that RNA editing can lead to the creation of a new exon in the human genome. Similarly to *Alu *retroposition and alternative splicing, RNA editing was not originally 'designed' to serve evolutionary purposes; it was rather recruited for this, probably serendipitously. This demonstrates that the creation of genomic novelty can be assisted by numerous molecular biological mechanisms, most of which were originally designed to function in other processes. The dynamism of our genome can, therefore, arise through surprising paths.

## Materials and methods

### Computational search for candidate edited *Alus*

ESTs and cDNAs from GenBank version 136 were aligned to the human genome (version hg16) to identify internal human exons that contain *Alu *elements, as described in [5]*Alu*-containing exons were identified using blastn analysis against the *Alu *consensus with a threshold of 1E-10. *Alu*s having AA/GT or AG/AT 3' ss/5' ss, which flank exons in the protein-coding region of the genes, were taken for further analysis. Only *Alu*-exons supported by multiple cDNAs and not containing stop codons (in the ESTs and cDNA) were further considered. Exons were manually screened to remove false computational predictions.

### Plasmid construction

Oligonucleotide primers were designed to amplify (from human genomic DNA) a minigene that contains exons 7, 8, and 9 (and the introns in between) of NARF (GenBank: NM_012336). Each primer contained an additional extension encoding a restriction enzyme sequence. The PCR product of NARF (2.8 kb) was restriction digested and inserted between the *Kpn*I/*Xho*I sites in the pEGFP-C3 vector, which contains green fluorescent protein (GFP; Clontech, Palo Alto, CA, USA).

### Site directed mutagenesis

Overlapping oligonucleotide primers containing the desired mutations were used to amplify a mutation-containing replica of the wild-type minigene plasmid, using *PfuTurbo *DNA polymerase (Stratagene, La Jolla, CA, USA) (Additional data file 3). After PCR amplification the reaction was digested with *Dpn*I restriction enzyme (New England Biolabs, Ipswich, MA, USA) for 1 h at 37°C, 1-3 μl of the reaction was transformed into the *Escherichia coli *XL-1 strain, and colonies were picked for Mini-prep extraction (Qiagen, Valencia, CA, USA). All plasmids were confirmed by sequencing.

### Transfection, RNA isolation and RT-PCR amplification

293T cells were cultured in Dulbecco's modified Eagle's medium, supplemented with 4.5 g/ml glucose (Biological Industries, Bait-Haemek, Israel), 10% fetal calf serum, 100 U/ml penicillin, 0.1 mg/ml streptomycin, and 1 U/ml nystatin (Biological Industries, Bait-Haemek, Israel). Cells were grown on 6-well plates under standard conditions at 37°C with 5% CO_2_. Cells were grown to 60% confluence, and transfection was performed using FuGENE6 (Roche Diagnostics, Basel, Switzerland) with 1 μg of plasmid DNA. Cells were harvested after 48 h. Total RNA was extracted using TRIzol Reagent (Sigma-Aldrich, St. Louis, MO, USA), followed by treatment with 1 U of RNase-free DNase (Ambion, Austin, TX, USA). Reverse transcription (RT) was preformed on 2 μg total RNA for 1 h at 42°C using Oligo-dT reverse primer and 2 U of reverse transcriptase avian myeloblastosis virus (A-AMV; Roche Diagnostics, Basel, Switzerland).

The spliced cDNA products derived from the expressed minigenes were detected by PCR using the pEGFP-C3-specific reverse primer and an exon 7 forward primer (Additional data file 3). Amplification was performed for 30 cycles, consisting of 1 minute at 94°C, 45 s at 61°C, and 1.5 minutes at 72°C. The products were resolved on 1.5% agarose gel and confirmed by sequencing. Band quantification was performed by densitometry scanning of ethidium bromide stained gels, using ImageJ software [47].

For the NMD treatment, cells 48 h post-transfection were subjected to 100 μg/ml puromycin (Sigma-Aldrich, St. Louis, MO, USA) for 4 h before RNA extraction.

### Analysis of RNA editing

Products from RT-PCR or from PCR obtained from commercial cDNAs (BioChain, Hayward, CA, USA) were separated by electrophoresis on 1.5% agarose gels. The appropriate PCR product was excised and the DNA was extracted and purified (Promega, Madison, WI, USA). Direct sequencing from both ends was done using the ABI PRISM (Applied Biosystems, Foster-City, CA, USA). The editing percentage from direct sequencing was calculated as GA+G×100
 MathType@MTEF@5@5@+=feaafiart1ev1aaatCvAUfeBSjuyZL2yd9gzLbvyNv2Caerbhv2BYDwAHbqedmvETj2BSbqee0evGueE0jxyaibaiKI8=vI8tuQ8FMI8Gi=hEeeu0xXdbba9frFj0=OqFfea0dXdd9vqai=hGuQ8kuc9pgc9s8qqaq=dirpe0xb9q8qiLsFr0=vr0=vr0dc8meaabaqaciGacaGaaeqabaqadeqadaaakeaadaWcaaqaaiaadEeaaeaacaWGbbGaey4kaSIaam4raaaacqGHxdaTcaaIXaGaaGimaiaaicdaaaa@3AC3@ for the forward primer and CT+C×100
 MathType@MTEF@5@5@+=feaafiart1ev1aaatCvAUfeBSjuyZL2yd9gzLbvyNv2Caerbhv2BYDwAHbqedmvETj2BSbqee0evGueE0jxyaibaiKI8=vI8tuQ8FMI8Gi=hEeeu0xXdbba9frFj0=OqFfea0dXdd9vqai=hGuQ8kuc9pgc9s8qqaq=dirpe0xb9q8qiLsFr0=vr0=vr0dc8meaabaqaciGacaGaaeqabaqadeqadaaakeaadaWcaaqaaiaadoeaaeaacaWGubGaey4kaSIaam4qaaaacqGHxdaTcaaIXaGaaGimaiaaicdaaaa@3ACE@ for the reverse primer; the presented percentages represent an average of three separated experiments or three independent amplifications. The nucleotides were quantified by the Discovery Studio (DS) Gene 1.5 program (Accelrys Inc., San Diego, CA, USA).

## Additional data files

The following additional data are available with the online version of this paper. Additional data file 1 contains two figures showing semi-quantitative PCR analysis. Additional data file 2 contains alignment of *Alu*Sx between human, chimpanzee and rhesus macaque, and also the rhesus macaque sequence of *Alu*Sx and its upstream surrounding *Alu*Y sequence. Additional data file 3 is a table listing the primer sequences used in this research.

## Supplementary Material

Additional data file 1 Two figures showing semi-quantitative PCR analysis Click here for file

Additional data file 2 Alignment of *Alu*Sx between human, chimpanzee and rhesus macaque, and the rhesus macaque sequence of *Alu*Sx and its upstream surrounding *Alu*Y sequence Click here for file

Additional data file 3 Primer sequences used in this research Click here for file

## Figures and Tables

**Table 1:** Exonic regulatory sequences predicted to be changed following editing in the five exonic sites

	Site e1*	Site e2*	Site e3*	Site e4*	Site e5*
SF2/ASF^†^		C**A**CACCT (3.2/3.6)			CTC**A**GGA (4.8/NA)
SC35^†^			**A**ATCACAG (NA/4)	A**A**TCACAG (NA/2.6)	
SRp40^†^		GC**A**CACC (2.6/NA)			CTACTC**A **(NA/5.1)
					ACTC**A**GG (4.2/NA)
SRp55^†^		TGC**A**CA (NA/3.1)			
Ast^‡^	**A**GTGC**A**^§^				TC**A**GGA
Burge^¶¥^	TGGT**G**G				**G**GGAGG
	GGT**A**GT				TC**A**GGA
hnRNP F					**G**GGA

The sequence of the exonic splicing regulatory sequence is shown. The edited 'A' nucleotide is in bold (unless otherwise indicated). The numbers in parentheses indicate the binding score before/after editing; NA indicates no available score. *Edited sites: E1, position 19 in the *Alu*-exon; E2, position 24; E3, position 32; E4, position 33; E5, position 46. ^†^Predicted by ESEfinder [31]. ^‡^Predicted by Goren *et al*. [28]. ^§^This unedited ESR overlaps with both E1 and E2 sites. ^¶^Predicted by RESCU-ESE [30]. ^¥^Sites E1 and E5 created two different hexamers for the edited and unedited position, according to RESCU-ESE [30].
