# Supplementary material for: RNA-editing-mediated exon evolution
Source: Genome Biol. 2007 Feb 27;8(2):R29. doi: 10.1186/gb-2007-8-2-r29 (PMC1852406; doi:10.1186/gb-2007-8-2-r29)
Supplement: Additional data file 1 — Two figures showing semi-quantitative PCR analysis [file gb-2007-8-2-r29-S1.doc]

**Additional file 1. Semi-quantitative PCR**. **A)** The PCR was preformed by making a mixture of all reagents and adding an equal amount of human brain cDNA (2.5ng/μl). To examine if the lower PCR product (exon skipping) was amplified to saturation after 30 cycles, we repeated the PCR analysis for 22, 24, 26, 28, 30, and 32 cycles. We ran the human brain cDNA from the different PCR cycles on the same 1.5% agarose gel. The PCR products were quantified using ImageJ. The lower PCR product starts to appear in cycle 24, and the intensity of the product increases exponentially between cycle 26, 28 and 30, whereas in cycle 32 the PCR product is saturated. The upper PCR product (exon inclusion) is detected in cycle 26 and shows an exponential increase in cycles 28, 30 and a lesser increase in cycle 32. **B)** A graphic presentation of the PCR products quantification. X axis is the number of PCR cycles; Y axis is the amount of pixels calculated by ImageJ. Lower and upper bands are in pink and blue curve, respectively. Similar results were obtained from two experiments.

**Additional file 1. A dilution of the cDNA used for the semi-quantitative PCR**. **A)** The PCR was preformed following a dilution of the cDNA (1.25ng/μl) and showed similar results as in Figure S1. To examine if the lower PCR product was amplified to saturation after 30 cycles we repeated the PCR analysis for 22, 24, 26, 28, 30, and 32 cycles. We ran the human brain cDNA from the different PCR cycles on the same 1.5% agarose gel. The PCR products were quantified using ImageJ. The lower PCR product (exon skipping) starts to appear in cycle 26, and the intensity of the product increases exponentially between cycle 28 30, and 32. The upper PCR product (exon inclusion) is detected in cycle 28 and shows an exponential increase in cycles 28, 30 and 32. However, even in this case the lower product in cycle 30 does not show saturation of the PCR product. **B)** A graphic presentation of the bands' quantification. X axis is the number of PCR cycles; Y axis is the amount of pixels calculated by ImageJ. Lower and upper bands are in pink and blue curve, respectively. Similar results were obtained from two experiments.
