# Supplementary material for: RNA-editing-mediated exon evolution
Source: Genome Biol. 2007 Feb 27;8(2):R29. doi: 10.1186/gb-2007-8-2-r29 (PMC1852406; doi:10.1186/gb-2007-8-2-r29)
Supplement: Additional data file 2 — Alignment of AluSx between human, chimpanzee and rhesus macaque, and the rhesus macaque sequence of AluSx and its upstream surrounding AluY sequence [file gb-2007-8-2-r29-S2.rtf]

Additional file 2: Human/Chimp/Rhesus alignment of NARF AluSx:

Editing sites	             1    2       34            5			
Human  tacaaaaattagccaggcgtggtagtgcacacctgtaatcacagctactcaggaggctgcggcaagagaatca-----
Chimp  tacaaaaattagccaggcgtggtagtgcacacctgtaatcacagctactcaggaggctgcggcaagagaatca-----
Rhesus agacctgtctggccgggtgtggtggtgcacacctataatcccagctactcgggaggctgaggcaagagaatcgcttga
                * *** ** ***** ********** ***** ********* ******** ************ 
Human  ---cttgaacccgggaggcagaggttacagcgagacaagattgcaccactggactccagcctgggcggcggaggt
Chimp  ---cttgaacccgggaggcagaggttacagcgagacaagattgcaccactggactccagcctgggcggcggaggt
Rhesus acccttgaacccgggaggcagaggttacagtgagccaagatcgcaccactgtactccagcctgggcggcggaggt
          *************************** *** ****** ********* ***********************

Alignment of AluSx between Human, Chimpanzee and Rhesus. The editing sites are marked by numbers at the upper part of the alignment. The 3'ss and 5'ss dinucleotides are marked in red. Fully conserved nucleotides are indicated by * at the lower part of the alignment. The E1, as well as the E5 editing sites in the Rhesus macaque (but not in chimpanzee) genome are encoding for “G” (highlighted in yellow), thus, presenting only the edited version of the gene in those sites.


Additional file 2: Rhesus sequence of AluSx:

aaaaattagccgggcgtggtggcggcgcctgtagtcccagctactcgggaggctgaggcaggagaatggcgggaacccgggaggcggagcttgcagtgagccgagatcgcgccactgcactccagcctgggcgacagagcgagactctgcctcaaaaaaaaaaaaaaaaaaaaaaatgggctgggcacagtggctcacacttgtaatcccagcacttcaggaggctgaggcaggctggtcacctgaggtcaggaggtcgagacctgtctggccaacatggtgaaaccccgtctctactaaaaatacaaaaattagccgggtgtggtggtgcacacctataatcccagctactcgggaggctgaggcaagagaatcgcttgaacccttgaacccgggaggcagaggttacagtgagccaagatcgcaccactgtactccagcctgggcggcggaggtgagactccatctcaaaaaaaaaaaaatg

In rhesus there was insertion of AluY (in the sense orientation, marked in green) upstream of the AluSx (highlighted in gray). In addition there was a deletion in the AluSx of 44 nucleotides (mark in red). Those 44 nucleotides deletion contain the human 3'ss (marked in bold and underlined), and also an insertion of 8 nucleotides (highlighted in light blue).
