# Supplementary material for: RNA-editing-mediated exon evolution
Source: Genome Biol. 2007 Feb 27;8(2):R29. doi: 10.1186/gb-2007-8-2-r29 (PMC1852406; doi:10.1186/gb-2007-8-2-r29)
Supplement: Additional data file 3 — Primer sequences used in this research [file gb-2007-8-2-r29-S3.rtf]

Additional file 3: Primers used throughout the article:

Name of primer	Sequence	
F-pEGFP-C3	CGGCATGGACGAGCTGTAC	
R-pEGFP-C3	CCTCTACAAATGTGGTATGGC	
F-NARF-exon 7	AACCTGTCTCCAGAGAAGATTTTCCACG	
R-NARF-exon 9	GAGTGTCGACGGCAGCATCTCTCAC	
F-Alu-antisense-delete	CGACCTTGTTTCTTAAAAAAAAAAGGG	
R-Alu-antisense-delete	CGAGGTCTCACTGTGTTGCCCAGC	
F-mut(A-G)-E1	ATTAGCCAGGCGTGGTGGTGCACACCTGTAATCAC	
R-mut(A-G)-E1	GTGATTACAGGTGTGCACCACCACGCCTGGCTAAT	
F-mut(A-G)-E2	CAGGCGTGGTAGTGCGCACCTGTAATCACAGC	
R-mut(A-G)-E2	GCTGTGATTACAGGTGCGCACTACCACGCCTG	
F-mut(A-G)-E3	GGTAGTGCACACCTGTGATCACAGCTACTCAGG	
R-mut(A-G)-E3	CCTGAGTAGCTGTGATCACAGGTGTGCACTACC	
F-mut(A-G)-E4	GTAGTGCACACCTGTAGTCACAGCTACTCAGGAG	
R-mut(A-G)-E4	CTCCTGAGTAGCTGTGACTACAGGTGTGCACTAC	
F-mut(A-G)-E5	CTGTAATCACAGCTACTCGGGAGGCTGCGGCAAGAG	
R-mut(A-G)-E5	CTCTTGCCGCAGCCTCCCGAGTAGCTGTGATTACAG	
F-mut(T-C)-near-E1	TTAGCCAGGCGTGGCAGTGCACACCTGTA	
R-mut(T-C)-near-E1	TACAGGTGTGCACTGCCACGCCTGGCTAA	
F-mut(G-C)-near-E1	TTAGCCAGGCGTGGTACTGCACACCTGTAATCAC	
R-mut(G-C)-near-E1	GTGATTACAGGTGTGCAGTACCACGCCTGGCTAA	
F-mut-antisense(A-G)-E1	GATTACAGGTGCGCCCGACCACACCAGGCTAAT	
R-mut-antisense(A-G)-E1	ATTAGCCTGGTGTGGTCGGGCGCACCTGTAATC	
F-create-3'ss	CGTCTCTACTAAAAATACAGAAATTAGCCAGGCGTGGTAG	
R-create-3'ss	CTACCACGCCTGGCTAATTTCTGTATTTTTAGTAGAGACG	
F-eliminate-3'ss	CGTCTCTACTAAAAATACATAAATTAGCCAGGCGTGGTAG	
R-eliminate-3'ss	CTACCACGCCTGGCTAATTTATGTATTTTTAGTAGAGACG	

·	Normal PCR amplifications were done for 30 cycles.
·	Site directed mutagenesis were done for 18 cycles.
